# Primary idiopathic osteoarthropathy

**DOI:** 10.11604/pamj.2017.28.88.13295

**Published:** 2017-09-29

**Authors:** Dhia Kaffel, Hend Riahi

**Affiliations:** 1 Rheumatology Department, Kassab Institute, Manouba, Tunisia; 2 Radiology Department, Kassab Institute, Manouba, Tunisia

**Keywords:** Primary idiopathic osteoarthropathy, digital clubbing, hippocratic fingers, periostosis

## Image in medicine

A 49-year-old man presented with arthralgias and effusion of the knees. His past medical syndrome revealed digital clubbing for 17 years (A) and an idiopathic Raynaud syndrome. Radiographs of the tibias and fibulas showed bilateral, symmetric, extended and multilayered periosteal thickening (B). Thoracic CT and cardiac ultrasound were normal, as well as inflammatory, hepatic and renal tests. Infectious investigations were negative. The diagnosis of primary idiopathic osteoarthropathy (HOA) was made with 3-year follow-up. HOA is a syndrome characterized by three clinical features: digital clubbing (also termed Hippocratic fingers), periostosis of tubular bones, and synovial effusions. HOA can be a primary entity, or can be secondary to extra skeletal conditions. Primary HOA is a rare disease, with male predilection. Hippocratic fingers show a distinctive bulbous deformity of the fingertips. It can be associated with large joint’s effusions but there is neither synovial membrane hypertrophy nor inflammatory cell exudation. Radiographs may show an acro-osteolysis, periostosis of tubular bones. The tibia, fibula, radius, and ulna are the most commonly affected bones.

**Figure 1:**
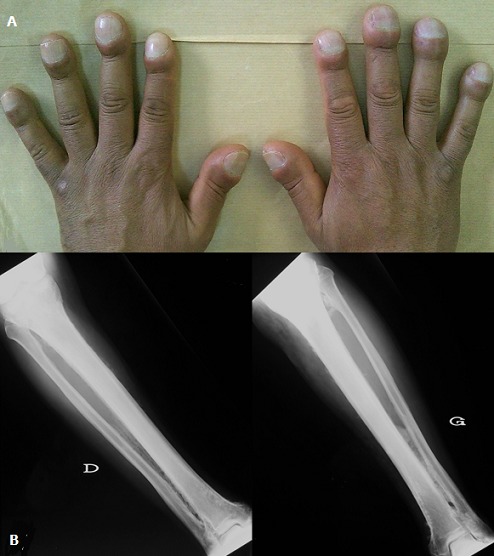
A) digital clubbing (hippocratic fingers); B) radiographs of the tibias and fibulas show bilateral extended and multilayered periosteal thickening

